# Identification of gene signatures for COAD using feature selection and Bayesian network approaches

**DOI:** 10.1038/s41598-022-12780-7

**Published:** 2022-05-24

**Authors:** Yangyang Wang, Xiaoguang Gao, Xinxin Ru, Pengzhan Sun, Jihan Wang

**Affiliations:** 1 School of Electronics and Information, Northwestern Polytechnical University, 1 Dongxiang Road, Xi’an, 710129 Shaanxi China; 2 Xi’an Key Laboratory of Stem Cell and Regenerative Medicine, Institute of Medical Research, Northwestern Polytechnical University, 127 West Youyi Road, Xi’an, 710072 Shaanxi China

**Keywords:** Cancer, Machine learning, Predictive markers

## Abstract

The combination of TCGA and GTEx databases will provide more comprehensive information for characterizing the human genome in health and disease, especially for underlying the cancer genetic alterations. Here we analyzed the gene expression profile of COAD in both tumor samples from TCGA and normal colon tissues from GTEx. Using the SNR-PPFS feature selection algorithms, we discovered a 38 gene signatures that performed well in distinguishing COAD tumors from normal samples. Bayesian network of the 38 genes revealed that DEGs with similar expression patterns or functions interacted more closely. We identified 14 up-DEGs that were significantly correlated with tumor stages. Cox regression analysis demonstrated that tumor stage, STMN4 and FAM135B dysregulation were independent prognostic factors for COAD survival outcomes. Overall, this study indicates that using feature selection approaches to select key gene signatures from high-dimensional datasets can be an effective way for studying cancer genomic characteristics.

## Introduction

Cancer is a major public health burden around the world, and it is the second leading cause of death in the United States^1^. According to the most recent American Cancer Society statistics for 2021, colon and rectum cancer (CRC) ranks the third in incidence and the third leading cause of cancer-related death worldwide. CRC remains one of the most common malignant tumors in the digestive system, and the type of colon adenocarcinomas (COAD) accounting for 95% of all cases of colon cancer^2^.

Cancers are well understood to be caused by genetic abnormalities in the target cells. In general, acquired mutations and epigenetic changes can influence tumor cell chromatin architecture and gene expression levels. As a result, identifying specific genetic markers that will promote molecular diagnosis and precision medicine in cancer is one of the most important aspects of cancer research. The Cancer Genome Atlas (TCGA, https://www.cancer.gov/tcga) program, an invaluable resource of cancer genomics, provides publicly available datasets for the development of improved methods for cancer diagnosis, treatment, and prevention^3,4^. The TCGA program molecularly characterizes over 20,000 primary cancer and matched normal samples spanning 33 cancer types, including COAD. Another human genomics project, the Genotype-Tissue Expression (GTEx, http://commonfund.nih.gov/GTEx), establishes a reference resource of gene expression from ‘normal’, disease-free tissues^5,6^. The GTEx project was established to characterize human transcriptomes within and across individuals for a wide range of primary tissues and cell types, including colon tissue^6^. Thus, combing the datasets from TCGA as tumor resources and GTEx as normal sample resources expands opportunities for data mining and deeper understanding of gene signatures in cancer research^7–9^.

Clinical diagnosis or prognosis prediction of cancer patients based on the high-throughput gene expression data depends greatly on the accuracy of disease classification. This necessitates the development of best classification models for cancer samples with high accuracy and low risk of misclassification. Gene expression data, such as RNA-sequencing or microarrays, usually suffer from the dimensionality problem: too many gene features and relative few samples. It is usually impractical to go through all of the features during the gene expression analysis. As a result, feature selection tends to be a prominent approach for disease classification, especially in datasets with a large number of features. It can eliminate relatively unimportant variables and improve classification accuracy and performance^10^. Wu et al.^11^ selected 300 biomarkers from 13,990 features with the combination of seven algorithms, including logistic regression and feature selection methods. A hybrid feature selection algorithm also has been used for searching optimal tumor biomarkers with significant performance for distinguishing tumor and normal samples^12^. The wavelet kernel ridge and radial basis kernel ridge regression were proposed to select the most relevant features which can be used for classification of microarray medical datasets^13^. Using a random forests model for feature selection, researchers identified a six-gene signature for predicting survival status in patients with head and neck squamous cell carcinoma (HNSCC) from the TCGA-HNSCC dataset^14^. Another five-gene signature (including RGS11, RGS10, RGS13, RGS4, and RGS3) has been identified as independent prognostic factors for ovarian cancer patients by using Lasso cox analysis^15^. In a study of melanoma, the feature selection approach was applied to discover and validate metastasis-related biomarkers based on single cell gene expression datasets^16^.

The current study aimed to identify gene signatures that could be used to classify COAD samples and normal colon tissues. Specifically, we established a feature selection model, SNR-PPFS, by combining the signal-to-ratio (SNR) ranking algorithm^17,18^ with the predictive permutation feature selection (PPFS) algorithm, a Markov blanket (MB) based feature subset selection method. The PPFS algorithm considers features both individually and collectively in order to provide the best set of features. Bioinformatic and biological analysis were also carried out to investigate the potential biological significance of the candidate genes identified through feature selection approaches. We anticipate that our research will provide a novel methodological foundation for the identification of COAD biomarkers as well as other cancer types.

## Methods and materials

### Data acquisition

**Figure 1:**
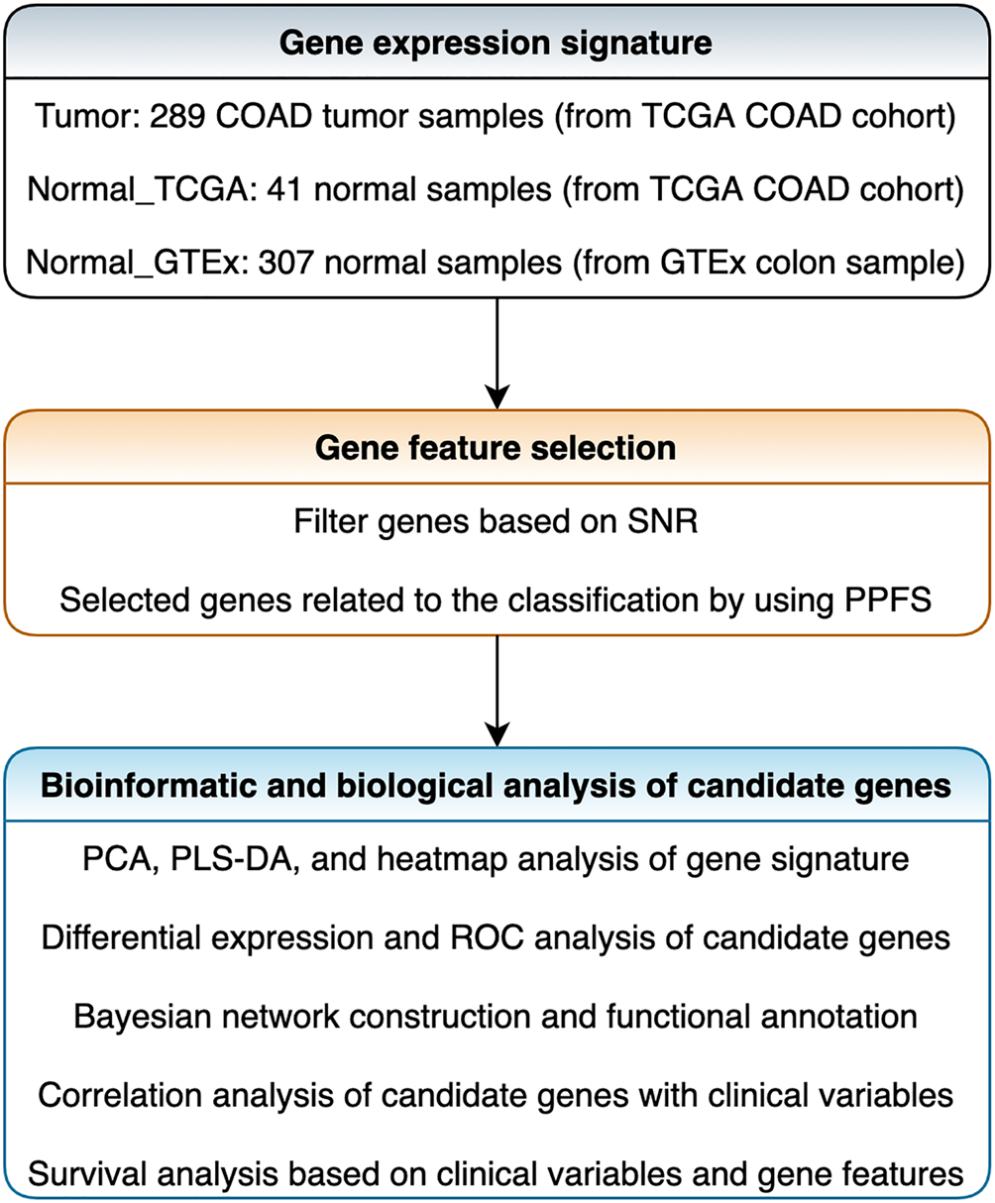
Overview of the study design.

Figure 1 depicted an overview of the study design. The datasets for a combined cohort of TCGA, TARGET, and GTEx samples were obtained from the UCSC xena website^19^. Firstly, the total RSEM expected_count (DESeq2 standardized) dataset was downloaded as the total gene expression profiling, which containing 19,039 bio-samples from both tumors and normal tissues (https://toil-xena-hub.s3.us-east-1.amazonaws.com/download/TCGA-GTEx-TARGET-gene-exp-counts.deseq2-normalized.log2.gz). We then chose samples of COAD tumor and normal colon tissue (selection criteria: for tumor tissue, primary_disease_or_tissue = “Colon Adenocarcinoma”; for normal tissue, primary_site = “Colon”) from the total gene expression dataset for the current study. Finally, 637 samples were recruited for research, including 289 COAD tumor samples (resourced from TCGA) and 348 normal samples. The 348 normal samples further contained 41 normal samples from the TCGA-COAD cohort and 307 normal colon tissues from GTEx. We also downloaded TCGA-COAD cohort’s phenotype and survival data for bioinformatic and biological analysis (phenotype data: https://gdc-hub.s3.us-east-1.amazonaws.com/download/TCGA-COAD.GDC_phenotype.tsv.gz; survival data: https://gdc-hub.s3.us-east-1.amazonaws.com/download/TCGA-COAD.survival.tsv). The clinicopathological characteristics of the 289 COAD tumor samples were summarized in Table 1.

**Table 1:** Clinical characteristics of COAD cases (data from the TCGA database), as well as Cox regression analysis of the clinical parameters.

Clinical characteristics	Number	Univariate analysis		Multivariate analysis	
		HR [95% CI]	*P*	HR [95% CI]	*P*
**Age (year)**					
< 65 (year)	126	1.00 [0.98–1.03]	0.841	1.02 [0.99–1.05]	0.251
≥ 65 (year)	161				
Not reported	2				
**Gender**					
Female	132	1.24 [0.64–2.40]	0.523	0.88 [0.43–1.77]	0.715
Male	155				
Not reported	2				
**Race**					
Asian	11	0.91 [0.62–1.35]	0.644	0.96 [0.64–1.45]	0.845
Black	55				
White	198				
Not reported	25				
**BMI**					
≤ 18.4	1	0.95 [0.90–1.01]	0.010	0.94 [0.89–1.00]	0.068
18.5–23.9	57				
24.0–27.9	61				
≥ 28	96				
Not reported	74				
**Tumor stage**					
Stage I	44	2.20 [1.45–3.33]	**0.0002**	2.39 [1.54–3.70]	** < 0.0001**
Stage II	110				
Stage III	83				
Stage IV	40				
Not reported	12				
**OS_status**					
Alive	213				
Dead	67				
Not reported	9				
**OS_time (day)**					
Alive	1001.28 ± 885.48				
Dead	776.99 ± 749.29				
Total number	289				

*BMI* body mass index, *OS* overall survival, *HR* hazard ratio, *CI* confidence interval.

Significant values are in bold.

### Gene feature selection using SNR-PPFS algorithms

After obtaining the gene expression dataset of 637 samples, we subsequently performed feature selection to identify gene signatures as classifier between tumor and normal groups. As shown in Fig. 1, the gene feature selection process consisted primarily of two steps, gene screening using the SNR algorithm and related gene selection using the PPFS method. All the steps were performed based on Python 3.8.

#### Screening genes using the SNR algorithm

SNR is an effective screening method that can quickly filter out genes that are unrelated to classification attributes. The expression is as follows. The numerator of the formula contains the average values of gene expression of the gene gi in the tumor and normal groups, and the denominator contains the standard deviations of the gene gi in the two groups. The higher the signal-to-noise ratio, the more important the gene is for classification.

$$SNR(g_{i} ) = \frac{{|u_{ + } (g_{i} ) - u_{ - } (g_{i} )|}}{{\delta_{ + } (g_{i} ) + \delta_{ - } (g_{i} )}}$$

#### Obtaining the Markov blanket genes using PPFS

##### The definition of Markov blanket

**Figure 2:**
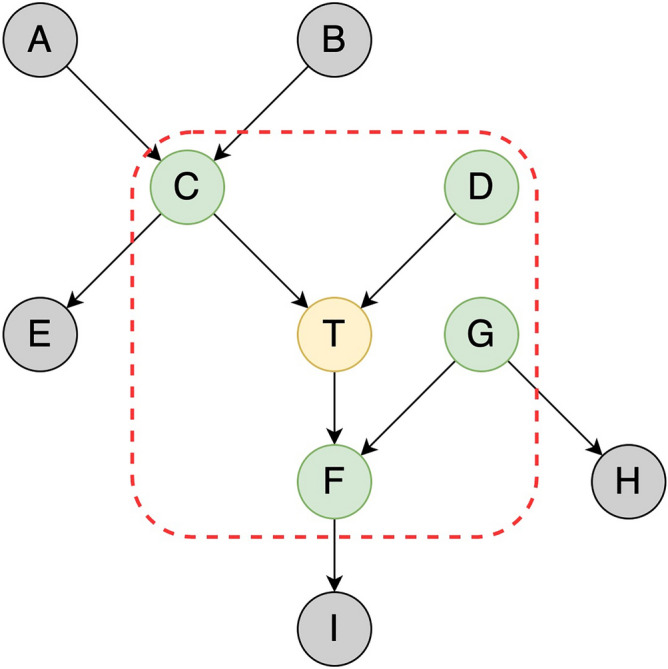
The diagram of an example of Markov blanket in a casual network. The T node with yellow color in the red rectangle is a target node, the other nodes form a Markov blanket of T node, and T node is independent of any node outside the rectangle.

Markov blanket is a widely used feature selection approach, which can be described as the following definitions and Fig. 2. It has already contained all the information related to the target node, and the non-Markov blanket nodes can be discarded safely to achieve the purpose of feature selection.

###### Definition 1

(*Markov condition*) Any variable (node) in a bayesian network is independent of its non-descendants given its parents.

###### Definition 2

(*Faithfulness*) Let G denote a Bayesian network. Let P denote a joint probability. G and P are said to be faithful to one another if all the conditional independencies entailed by G and the Markov condition is present in P.

###### Definition 3

(*Markov blanket*) Under the faithful condition, MB (Y) is the minimal set conditioned on which all other variables are independent of Y, i.e., (X\MB(Y)  Y|MB(Y)).

##### Predictive permutation feature selection

The PPFS^20^ is a Markov blanket theory-based feature selection algorithm that selects a subset of features based on their performance both individually and as a group. It can automatically decide how many features to take and try to find the optimal combination of features, especially it performs well on high-dimensional data. In this case, we combined the SNR and PPFS to obtain the final gene signatures for classifying tumor and normal samples; the procedures were detailed in Algorithm 1.
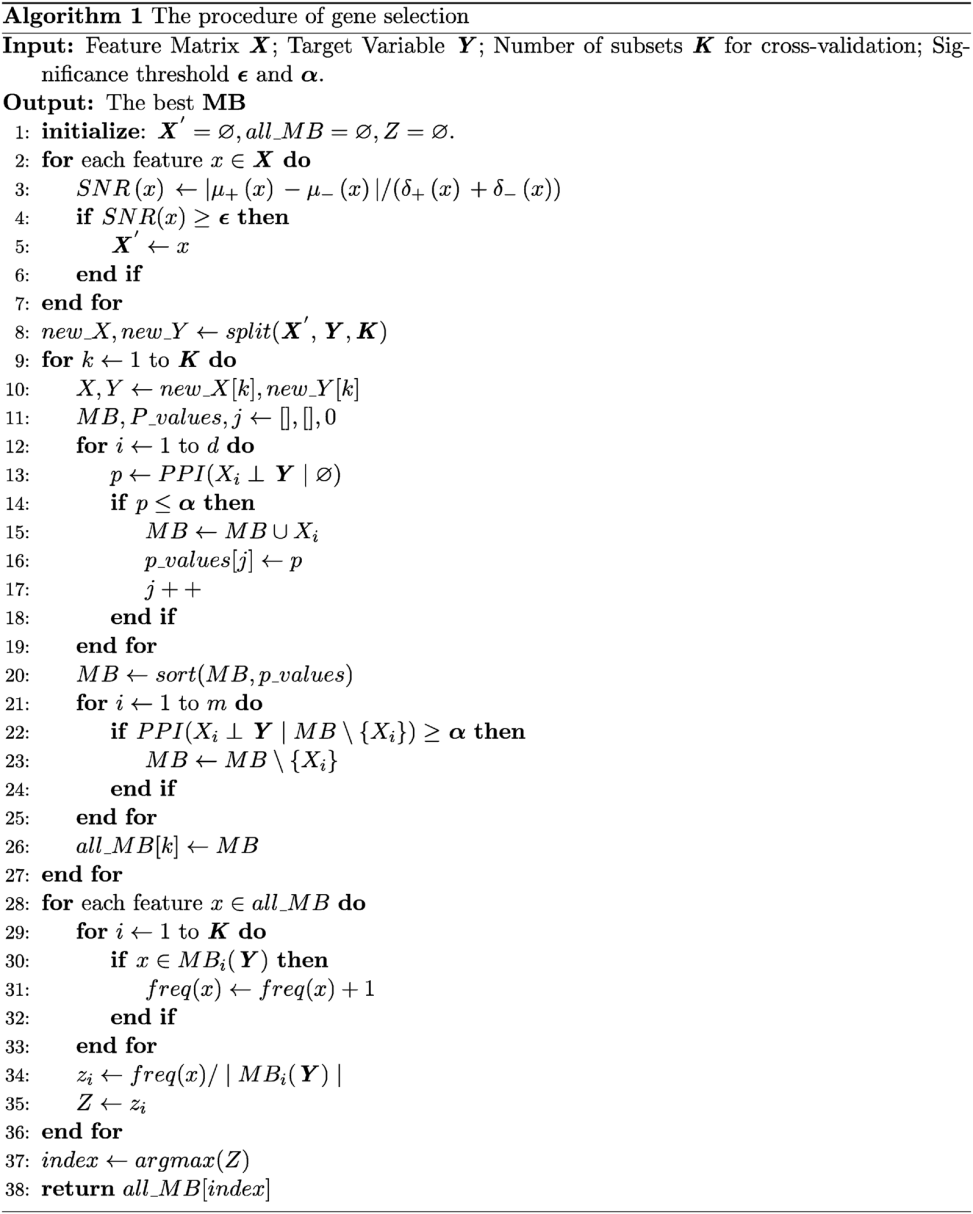


### Bioinformatic and biological analysis

#### Bayesian network and gene functional annotation

Following the feature selection, we will identify candidate genes in tumors. We then used the Bayesian structure learning algorithm of PCStable^21^ to construct a gene regulatory network, based on the expression profiles of the candidate genes. Furthermore, the protein–protein interaction (PPI) network and functional annotation were carried out using the online platform STRING: functional protein association networks (https://www.string-db.org/).

#### PCA, PLS-DA and heatmap analysis

We conducted principal component analysis (PCA), partial least squares discriminant analysis (PLD-DA) and heatmap analysis to illustrate the performance of classification between tumor and normal groups. Specifically, the PCA, PLS-DA and heatmap analysis were carried out in R using the pca function in “FactoMineR” package, the plsda function in “mixOmics” package, and the pheatmap function in “pheatmap” package, respectively, based on the candidate gene expression profiling of 637 samples.

#### Differential expression and ROC analysis of candidate genes

The R package “limma” was used to compare the expression of candidate genes in tumor and normal samples. To evaluate the performance of candidate genes in the diagnosis of COAD, the specificity, sensitivity, and area under the curve (AUC) values were obtained using receiver operator characteristic (ROC) analysis in MedCalc software.

#### Correlation analysis of candidate genes with the clinicopathological characteristics of COAD patients

We used Pearson correlation in R to examine the relationship between gene expression and clinicopathological characteristics of COAD patients, particularly tumor stage status. For survival analysis, R packages “survival” and “survminer” were applied. Both univariate and multivariate Cox regression analysis were performed to estimate the simultaneous effects based on the clinical parameters and candidate gene expression signature in COAD patients, with *P* < 0.05 as the statistically significant level. Kaplan–Meier survival curves of candidate genes were also visualized.

## Results

### Feature selection identified a 38 gene signatures for classifying COAD tumor and normal samples

We found some genes with an expression value of “0” during the pre-processing, and we filtered out those genes with the expression of “0” in more than two-thirds of the 637 samples to reduce the noise. Following data pre-processing, we obtained expression profiling of over 50,000 gene symbols for each of the 637 samples. We then conducted feature selection to determine the most valuable gene features in classifying tumor and normal groups. The SNR approach identifies expression patterns with the greatest difference in average expression between two groups and the least variation in expression within each group; genes can be ranked according to their expression levels using the SNR test statistic. In this study, we first screened a total of 430 gene signatures by SNR method. Further, the 430 genes were matched by PPFS algorithm. Finally, the best set of gene features containing 38 genes was identified for classification.

### Expression profiling analysis of the candidate 38 genes

**Figure 3:**
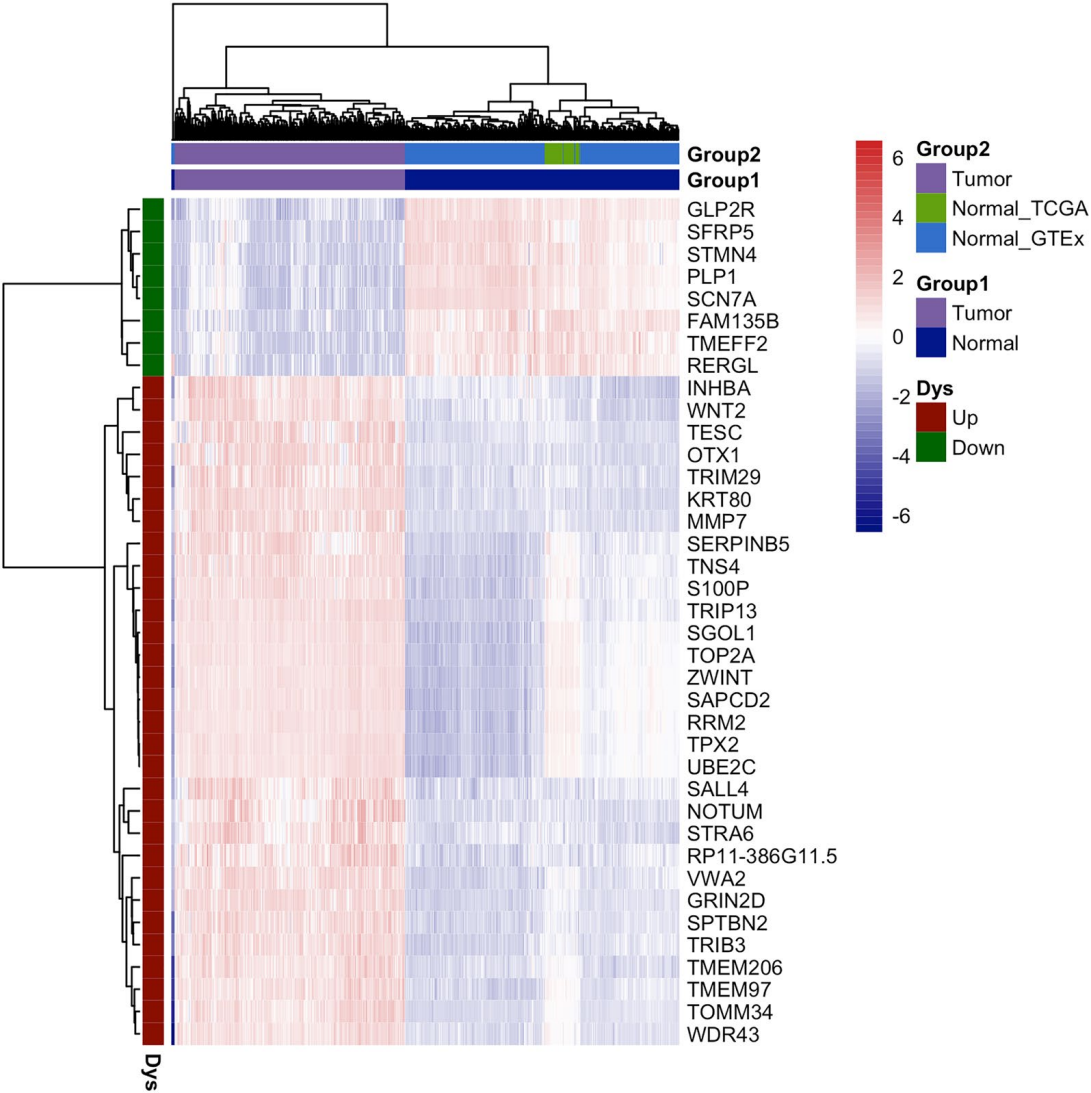
Bi-clustering analysis of the 38 genes that were screened using feature selection. The analysis was carried out in R using the “pheatmap” package. All of the samples were mainly divided into two groups: tumor and normal, with the latter including normal_TCGA and normal_GTEx subgroups. The samples and genes were represented by the horizontal and vertical axis, respectively.

Previously, 38 genes were identified as classifiers between tumor and normal samples through feature selection approach. To investigate the expression patterns of these 38 genes in COAD tumors and normal samples, differential expression analysis was performed using Limma method. Table 2 displayed the fold change and statistical level of the candidate genes in tumor versus normal groups, as well as the specificity, sensitivity, and AUC values in ROC analysis. The majority (30 out of 38 genes) of the differentially expressed genes (DEGs) were up-regulated in tumors, as shown in Table 2 and the heatmap in Fig. 3. In particular, all these 38 genes demonstrated promising discrimination power in distinguishing tumors from normal samples (specificity range: 90.5–99.7, sensitivity range: 90.0–99.7, AUC range: 0.954–0.998).

**Table 2:** Differential expression and ROC analysis of the 38 candidate DEGs.

DEGs	Differential analysis				ROC analysis		
	logFC	AveExpr(Tumor)	AveExpr(Normal)	adj.P.Val	Specificity	Sensitivity	AUC
MMP7	8.597	10.269	1.672	2.24E−247	98.9	97.2	0.994
KRT80*	7.498	10.622	3.124	1.62E−317	99.7	99.0	0.998
NOTUM*	7.363	8.637	1.274	2.09E−175	98.6	95.5	0.993
TNS4	6.979	12.162	5.183	9.91E−181	98.3	91.7	0.987
S100P	6.859	12.859	6.000	2.83E−162	91.7	96.5	0.978
SERPINB5*	6.676	10.504	3.829	1.35E−162	93.4	91.0	0.970
GRIN2D*	6.441	10.372	3.931	1.59E−267	96.8	98.3	0.996
UBE2C*	6.256	11.824	5.568	2.34E−138	96.0	97.2	0.993
RRM2	6.203	12.360	6.157	2.62E−128	93.1	98.6	0.987
SAPCD2	6.107	11.959	5.852	1.36E−134	96.3	95.5	0.992
VWA2	6.032	9.438	3.406	1.29E−227	96.8	95.5	0.993
TPX2	6.020	12.635	6.615	1.96E−149	95.4	98.3	0.994
WNT2	5.954	8.294	2.341	6.37E−216	98.9	93.8	0.993
TOP2A*	5.555	13.049	7.494	2.35E−141	94.8	96.5	0.991
STRA6	5.416	7.839	2.423	4.36E−175	98.3	91.3	0.981
OTX1	5.411	6.538	1.127	1.21E−222	98.9	93.4	0.985
TRIM29	5.307	11.420	6.113	1.93E−195	98.6	92.0	0.990
INHBA*	5.167	10.480	5.313	3.06E−167	96.8	92.4	0.982
SGOL1	5.057	8.763	3.707	1.11E−146	99.7	93.1	0.989
TRIB3*^#^	4.798	11.587	6.789	1.96E−218	97.1	98.6	0.996
TRIP13	4.755	10.238	5.484	1.35E−204	97.7	99.7	0.997
TESC	4.410	10.367	5.957	2.79E−162	95.1	90.7	0.954
ZWINT	4.361	11.312	6.951	5.32E−124	90.8	95.5	0.976
SALL4*	4.249	7.514	3.265	8.32E−158	96.8	90.7	0.972
SPTBN2*	4.032	10.730	6.699	5.31E−219	98.3	99.0	0.993
RP11-386G11.5*	3.577	5.779	2.202	7.02E−165	96.3	93.1	0.984
TMEM97	3.373	11.728	8.355	1.33E−164	96.8	95.5	0.992
TOMM34*	3.230	12.094	8.865	1.15E−167	99.4	93.1	0.991
TMEM206*	2.650	9.031	6.381	1.57E−180	98.9	96.2	0.996
WDR43*	2.578	11.841	9.263	6.30E−148	97.4	99.7	0.996
TMEFF2	− 3.729	1.225	4.954	2.34E−167	92.8	93.4	0.966
STMN4^#^	− 4.236	1.416	5.651	6.62E−161	94.5	91.3	0.962
FAM135B^#^	− 4.303	1.583	5.885	4.34E−165	96.0	90.3	0.966
GLP2R	− 4.818	4.532	9.349	2.26E−175	98.0	98.3	0.985
RERGL	− 4.894	1.734	6.628	4.72E−163	90.5	93.1	0.968
SFRP5	− 6.143	2.305	8.448	4.49E−164	96.0	90.0	0.964
SCN7A	− 6.757	3.803	10.560	2.82E−157	93.7	90.7	0.969
PLP1	− 7.039	3.251	10.290	4.04E−181	96.8	95.5	0.979

The differential analysis was performed by limma “package” in R. ROC analysis was carried out using MedCalc software. Genes with “*”showed the tumor stage-positive related genes (*P* < 0.05). Gene with “#”showed the survival-related genes (*P* < 0.05).

**Figure 4:**
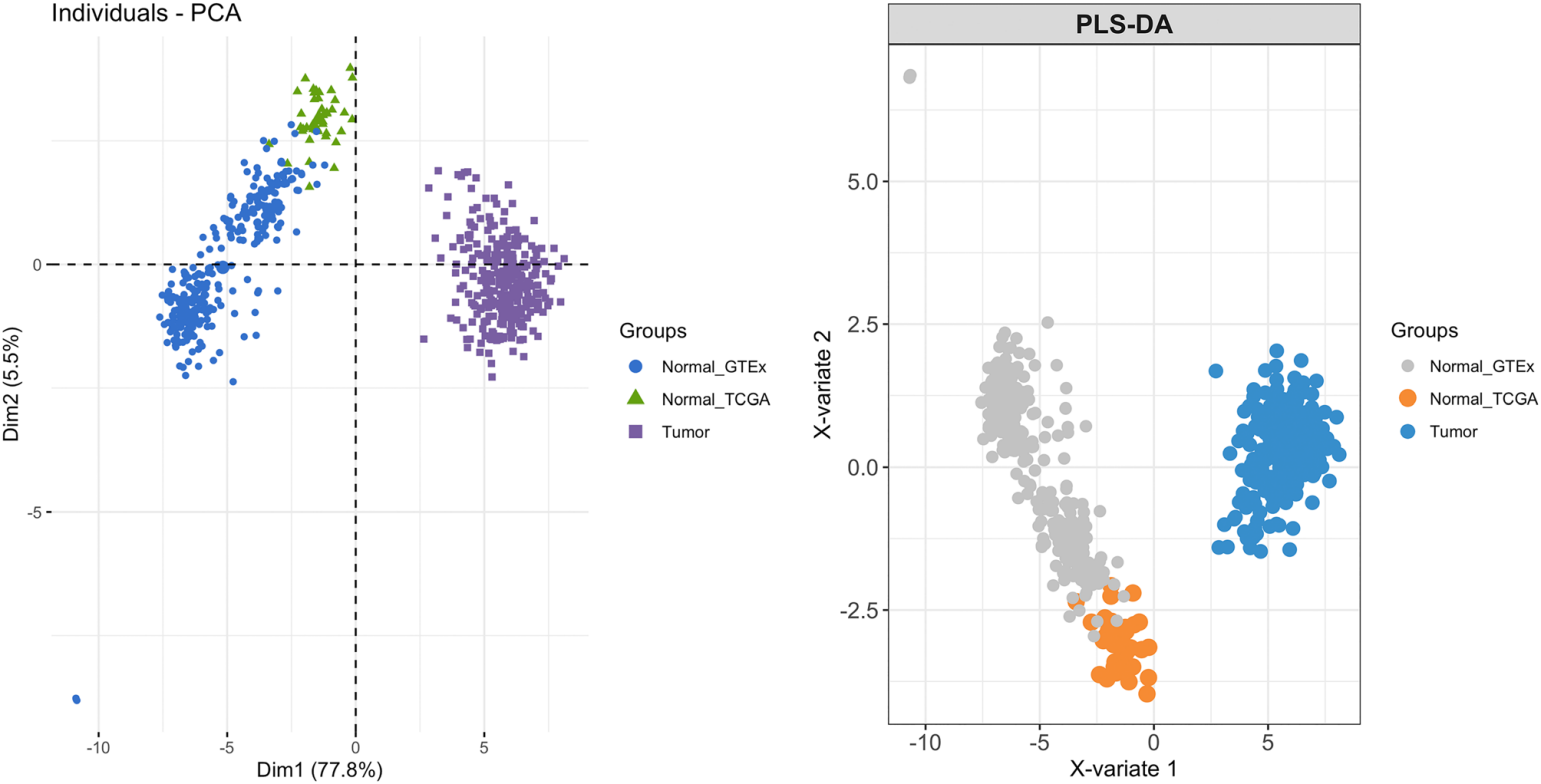
PCA and PLS-DA plot based on the expression pattern of the 38 genes. The analysis was performed using the “FactoMineR” package for PCA and the “mixOmics” package for PLS-DA in R. Each dot, triangle, and square represent a sample.

The heatmap, PCA and PLS-DA model of samples based on the 38 gene signatures were performed to visualize the clustering performance. As expected, fully separated models between tumor and normal samples were observed when performing PCA and PLS-DA (Fig. 4). In the current study, the normal group was further subdivided into two subgroups according to the sample source databases: normal-TCGA and normal-GTEx. Thus, we also took into account the information of subgroups when performing the clustering analysis. As shown in Figs. 3 and 4, the two subgroups of normal samples overlapped to a small extent, and both sets of normal samples could be completely separated with tumor samples.

### Using Bayesian network constructing gene regulatory network

**Figure 5:**
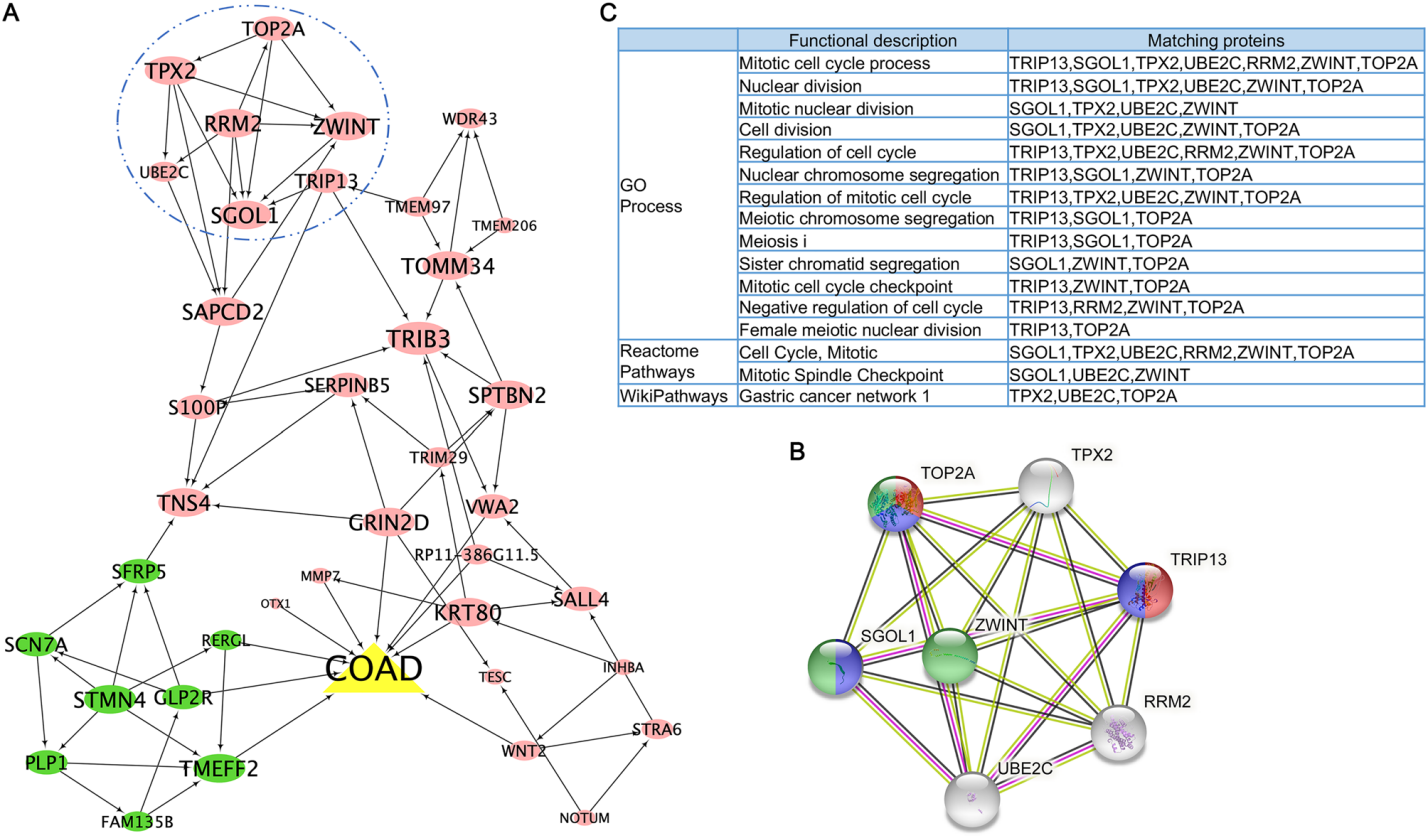
Bayesian network of the 38 candidate genes as well as the PPI network analysis. (**A**) Bayesian network of the 38 candidate genes. The red and green eclipses represent the up-regulated and down-regulated DEGs in COAD tumors, respectively. (**B**) PPI network of the seven up-DEGs [the seven genes in circle of (**A**] from STRING functional database. (**C**) Functional annotation of the genes in the PPI network.

In this study, we proposed using Bayesian network to construct gene regulatory networks for the 38 candidate genes based on their expression profiles. The 38 DEGs interacted with each other to some extent (Fig. 5A). Specifically, in this connected network, the eight down-DEGs interacted with the up-DEGs in relatively separate ways. Furthermore, we discovered that the Bayesian network aids in the discovery of biological gene-regulatory interactions. For instance, we identified seven up-DEGs interacting with each other in the Bayesian network (as shown in circle in Fig. 5A). Further, a complete protein–protein interaction (PPI) network was obtained from the STRING online platform based on the seven up-DEGs (Fig. 5B). Functional annotation of the PPI network was primarily involved in biological process related to cell cycle and nuclear division, as well as gastric cancer disease (Fig. 5C). These findings indicated that in a Bayesian network, genes with similar expression patterns and functions are tend to be closer in the connections, which will help bridge the gap between an individual gene and a system biological interpretation in the high throughput bioinformatics research.

### Correlation analysis of candidate genes and clinicopathological characteristics of COAD patients

**Figure 6:**
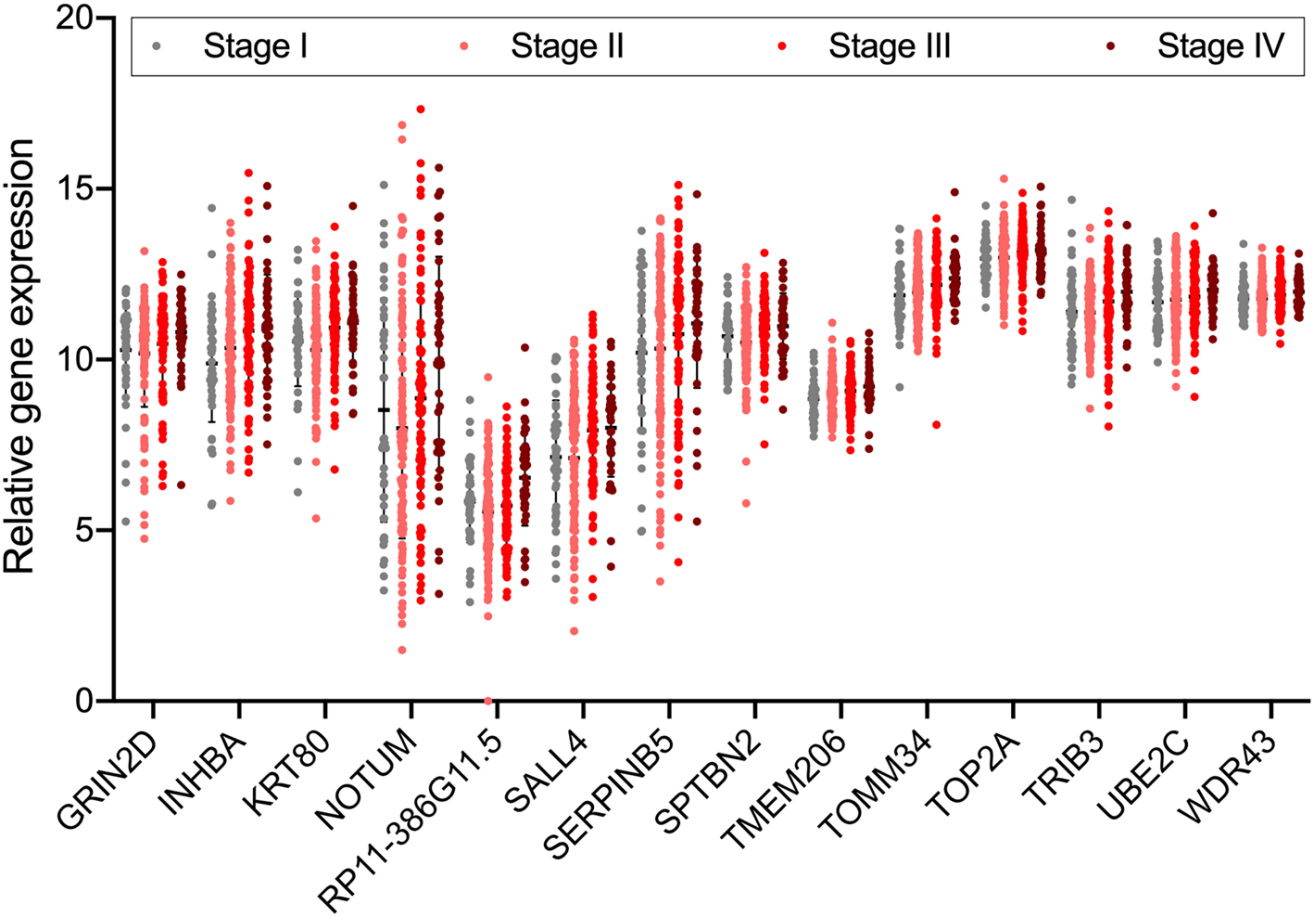
Relative gene expression plot of the 14 stage-positive related DEGs. GraphPad Prism was used to create the scatter plot, and each dot represents a sample.

The TCGA database contains relatively comprehensive clinicopathological information on tumor samples. We then investigated whether the candidate genes were related to the clinicopathological characteristics of COAD patients. As summarized in Table 1, the tumor samples could be divided into different subgroups based on basic clinical information such as age, gender, race, and body mass index (BMI). According to the PLS-DA model (Fig. S1), the 38-gene expression signature could not well distinguish different subgroups of tumor samples based on the above basic clinical information. While, from the 38 gene signatures, we identified 14 candidate genes that were positively related to tumor stage status (*P* < 0.05 in Pearson correlation). Figure 6 illustrated the relative expression of the 14 stage-positive related genes in tumor samples of different stages, and Table S1 and Fig. S2 summarized the correlation scatter plots, coefficient values and statistical levels of the Pearson correlation. What’s more, we found that the 14 DEGs were up-regulated in tumors compared to normal samples (Tables 1 and S1), implying that the stage related genes may help reflecting the tumor progression of COAD.

**Figure 7:**
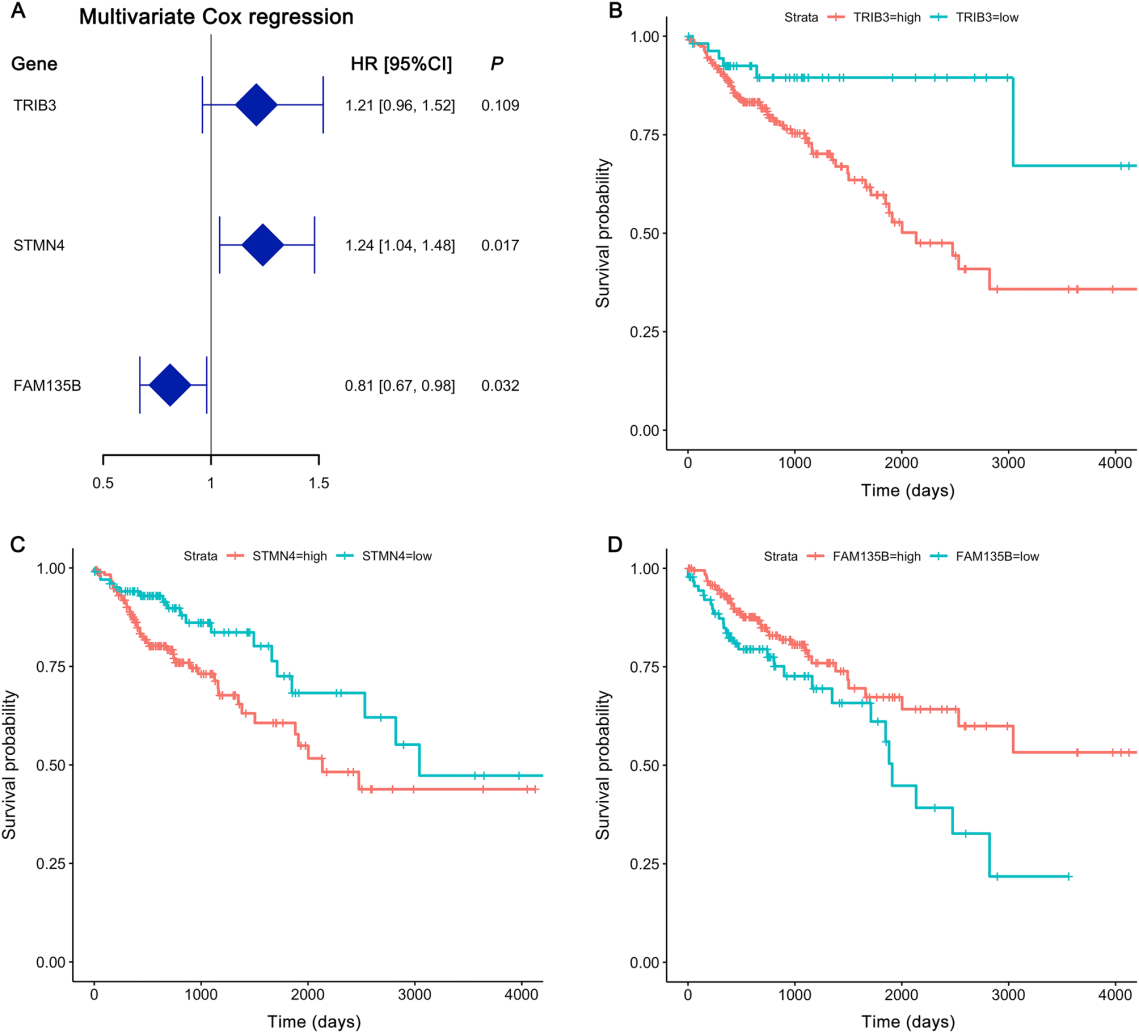
Multivariate Cox regression and Kaplan–Meier survival curves of three candidate DEGs. The analysis was carried out in R using the “survival” and “survminer” packages. (**A**) Multivariate Cox regression forest plot of the three candidate genes. HR: hazard ratio; CI: confidence interval. (**B**–**D**) Kaplan–Meier survival curves for TRIB3, STMN4, and FAM135B, respectively. The cut-off points divided gene expression values into high (high) and low (low) groups.

To investigate the prognostic factors for COAD, the Cox regression model for survival analysis was conducted. The risk score (HR > 1) was significantly positively correlated with tumor stage in both univariate and multivariate Cox regression analysis, indicating that it could be recognized as an independent risk factor for patients’ prognosis (Table 1 and Fig. S3). We also evaluated the effects of the 38 DEGs on survival outcomes. Overall, the expression pattern of 38 DEGs was not significantly correlated with the survival outcomes (*P* > 0.05) in univariate Cox regression analysis, as shown in Table S2. When we set the screening criteria to 0.05 < *P* < 0.1 as having an influential trend, then TRIB3, STMN4 and FAM135B were found to have survival correlations in univariate Cox regression analysis. The risk score was significantly correlated with the differential expression of STMN4 (HR > 1, *P* < 0.05) and FAM135B (HR < 1, *P* < 0.05) in multivariate Cox regression analysis of the three candidate genes, as summarized in Table S2 and Fig. 7A. The Kaplan–Meier survival curves also revealed that high TRIB3 and STMN4 expression was associated with a lower overall survival probability, whereas high FAM135B expression was a better survival outcome (Fig. 7B–D). Taken together, our suggested that STMN4 and FAM135B dysregulation are independent prognostic factors for COAD patients.

## Discussion

With the development of high-throughput techniques in biology and life sciences, more and more omics datasets are being generated, particular in the field of cancer research. In recent years, the application of GTEx project has greatly improved the ability to study the genomics of normal tissues or cell lines^22,23^, providing invaluable reference data for cancer studies of the corresponding tissues/organs. The feature selection approach helps to locate important and representative indicators from high-dimensional datasets, which is important for the advancement of precision medicine, such as cancer diagnosis and treatment. In our study, we utilized both SNR and PPFS methods before and after, and finally discovered a set of 38 genes with promising performance in distinguishing COAD tumors from normal colon tissues, based on the combining dataset from both the TCGA-COAD cohort and GTEx normal colon samples.

The Bayesian network (also known as causal network) is a directed acyclic graphical model developed in the late 1970’s. The nodes represent the variables and the linkages represent informational or causal dependencies among the variables in a Bayesian network. Bayesian networks are widely used for modeling and inferring gene regulatory networks in biological applications, which provides an efficient way to study functional genomes. Here we constructed a Bayesian network based on the 38-gene expression profiles and classification labels (tumor or normal). The differential analysis revealed that the majority of the 38 DEGs were up-regulated, with only eight DEGs being down-regulated in COAD tumors compared to normal colon tissues. Interestingly, the gene nodes in the Bayesian network tended to be initially clustered according to the expression pattern. Based on this hypothesis, we may be able to predict the expression changes of novel genes since DEGs with similar expression patterns are tend to cluster together in a Bayesian network. It’s also worth mentioning that Bayesian networks have been applied for inferring the structure of biological modules that reflect causal molecular mechanisms or statistical associations of the underlying system^24^. In this study, for example, a biologically meaningful STRING PPI network involving seven up-DEGs was identified in the 38-gene Bayesian network. The seven DEGs in the PPI-network were all up-regulated in COAD tumor samples and were mainly enriched in cell cycle and division-related functions. Cell cycle deregulation is well known to be one of the most frequent alterations during tumorigenesis and development^25,26^. Thus, the findings above support the theory that using Bayesian networks not only provides useful information for disease classification, diagnosis and prediction, but also guides in inferring the structure of biological meaningful modules. However, Bayesian network model is not that perfect when imitating gene regulatory network. Gene regulatory networks are bipartite, since two genes can regulate each other in a network. In response to causality, the Bayesian network only forms a unidirectional mode rather than a bidirectional mode, which does not accurately reflect the actual gene regulation situation. What’s more, when the number of features (for example, genes) is relatively large, it is difficult to construct a Bayesian network, which further supports the significance of gene feature selection when studying the high-throughput dataset.

ROC analysis of the 38 DEGs showed ideal diagnostic accuracy, specificity, and sensitivity for COAD tumor samples, supporting our hypothesis that feature selection aids in obtaining effective gene features in cancer research. More importantly, parts of the candidate genes were found to be significantly correlated with tumor stage and survival outcomes in COAD patients. Studies have shown that TOP2A played important roles in the tumorigenesis of many types of cancer, including colon cancer, and knockdown of TOP2A suppressed the proliferation and invasion of colon cancer cells^27^. Previously, DNA microarray and two-color FISH detection revealed that the ubiquitin-conjugating enzyme E2C gene (UBE2C) was significantly overexpressed in both primary tumors and liver metastases of colon cancer^28^. TOP2A and UBE2C were also found to be up-regulated in COAD tumors when compared to normal tissues in this study. Meanwhile, the two genes were found to be positively correlated with tumor stage and to be functionally enriched in the gastric cancer network, implying that they may function as oncogenes in gastrointestinal tumors. Similarly, other stage-related up-DEGs discovered in our study have also been reported in colon cancer researches. A recent bioinformatic analysis, for example, revealed that key genes such as GRIND, KRT80, and SPTBN2 have high diagnosis values in CRC patients^29^. Furthermore, high levels of KRT80 mRNA were also observed in CRC cell lines^30^. INHBA promoted the proliferation, migration, and invasion of colon cancer cells^31^, and has been shown to be a prognostic predictor for COAD patients^32^. SALL4 mRNA has been identified as a marker for the diagnosis of several cancers^33,34^. The anti-cancer effects of chrysin on tumor cells in colon cancer included induction of apoptosis and attenuation of the SALL4 expression^35^. It has also been proposed that SERPINB5 in CRC is associated with tumor location, poor histological differentiation, microsatellite instability, and poor prognosis^36^. TMEM206 was demonstrated to promote CRC malignancy by interacting with AKT and extracellular signal-regulated kinase signaling pathways^37^. A study showed that TOMM34 expression was elevated in the majority of human colon cancer samples, and the siRNA-TOMM34 approach effectively suppressed gene expression and significantly inhibited cell growth in colon cancer HCT116 cells^38^. Researchers identified several candidate cancer driver genes, including TOMM34, in both mRNA and protein levels in a proteogenomic study of human CRC samples^39^. NOTUM, one of the Wnt target genes, was found to be up-regulated in clinical specimens of colon cancer^40^. Similarly, immunohistochemistry detection confirmed WDR43 overexpression in CRC patient specimens^41^. What’s more, several studies have reported the oncogenic role of TRIB3 in CRC^42^. In intestine cells, TRIB3 interacts with β-catenin and TCF4 to increase the expression of genes associated with cancer stem cells and promote CRC tumorigenesis^43^. Approaches to inhibiting TRIB3 activity may be developed for cancer therapy^43^. In this research, we discovered a positive relationship between TRIB3 expression and tumor stage, and high levels of TRIB3 indicating a poorer survival. Furthermore, we discovered that the gene FAM135B, which had not previously been described in colon cancer, was down-regulated and served as a prognostic factor for COAD. Overexpression of FAM135B has been reported in esophageal squamous cell cancer (ESCC)^44^. The FAM135B/AKT/mTOR feedforward loop promoted ESCC progression^45^, and silencing FAM135B improved the radiosensitivity of esophageal carcinoma cell^46^. This phenomenon contradicts our findings that FAM135B was significantly down-expressed in COAD samples, which needs to be confirmed further. Despite this, we may conclude that feature selection can greatly help to identify key candidate genes in cancer research. The majority of the candidate genes have previously been reported, with the same alteration trend as our findings. While another relatively novel gene features can be obtained for specific cancer types, this will broaden the field of biomarker discovery service for tumor diagnosis and treatment, both technically and theoretically.

## Conclusions

In summary, we identified a 38 gene signatures with ideal performance when classifying COAD tumor from normal samples by using feature selection methods in this study. The majority of the 38 DEGs were significantly up-regulated in tumor samples compared to normal samples. In the Bayesian network, we found that genes with similar expression patterns or functions interacted more closely. Moreover, some of the candidate genes, such as TRIB3, KRT80, and FAM135B, were found to be correlated with tumor stage or survival outcomes, implying that these candidate genes could serve as promising prognostic biomarkers for COAD patients. Taken together, our study highlights the necessity and importance of feature selection approaches in cancer research, especially for high-dimensional datasets, which will significantly advance the development of precision medicine.

## Supplementary Information

Supplementary Information 1.

Supplementary Information 2.

Supplementary Information 3.

Supplementary Information 4.

Supplementary Information 5.

Supplementary Information 6.

## Data Availability

The raw data of this study have been deposited in FigShare (https://figshare.com/) with the link: https://doi.org/10.6084/m9.figshare.19093307.
