# Supplementary figures and images for: Identification of gene signatures for COAD using feature selection and Bayesian network approaches
Source: Sci Rep. 2022 May 24;12:8761. doi: 10.1038/s41598-022-12780-7 (PMC9130243; doi:10.1038/s41598-022-12780-7)

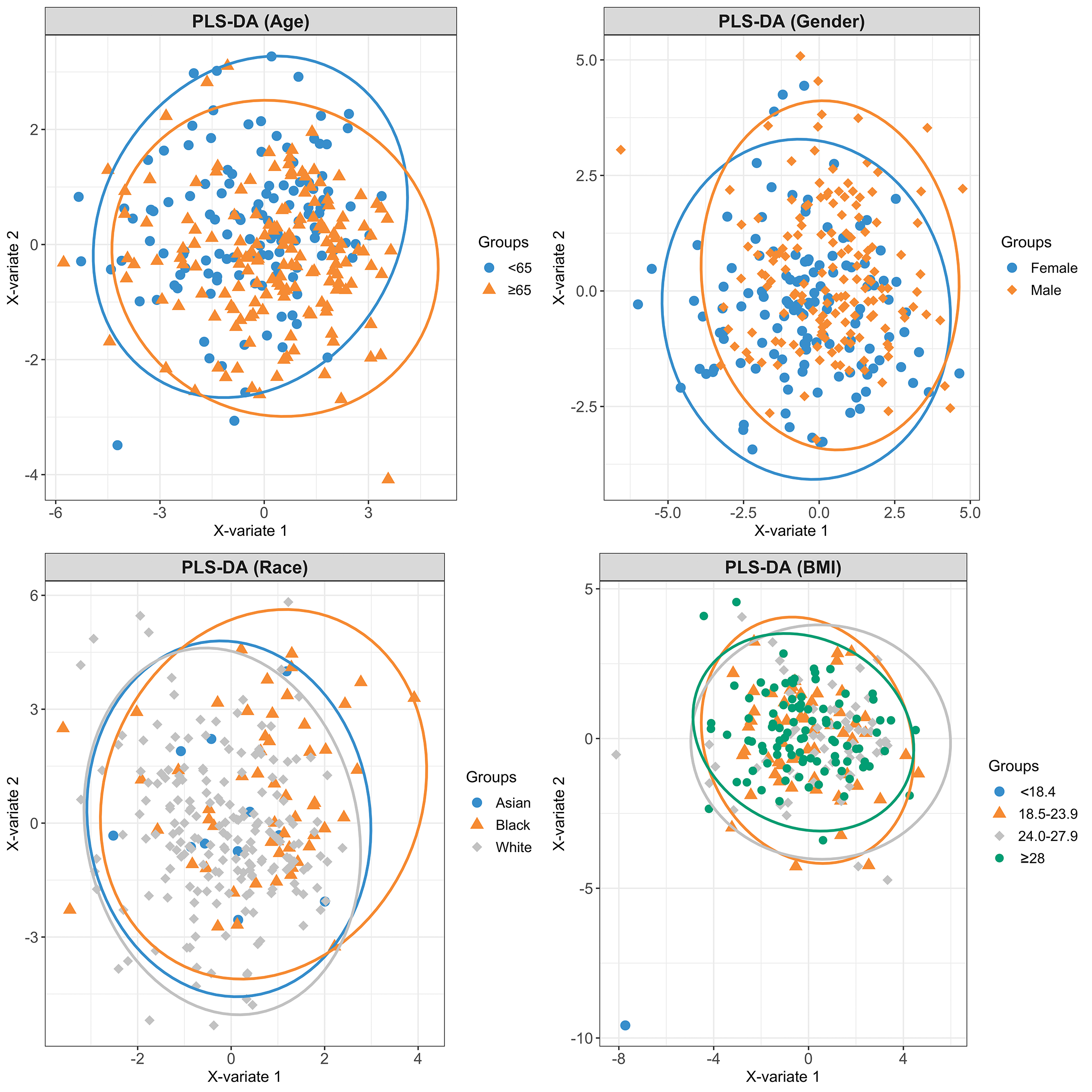

Supplement: Supplementary file 1 — Supplementary Information 1. [file 41598_2022_12780_MOESM1_ESM.tif]

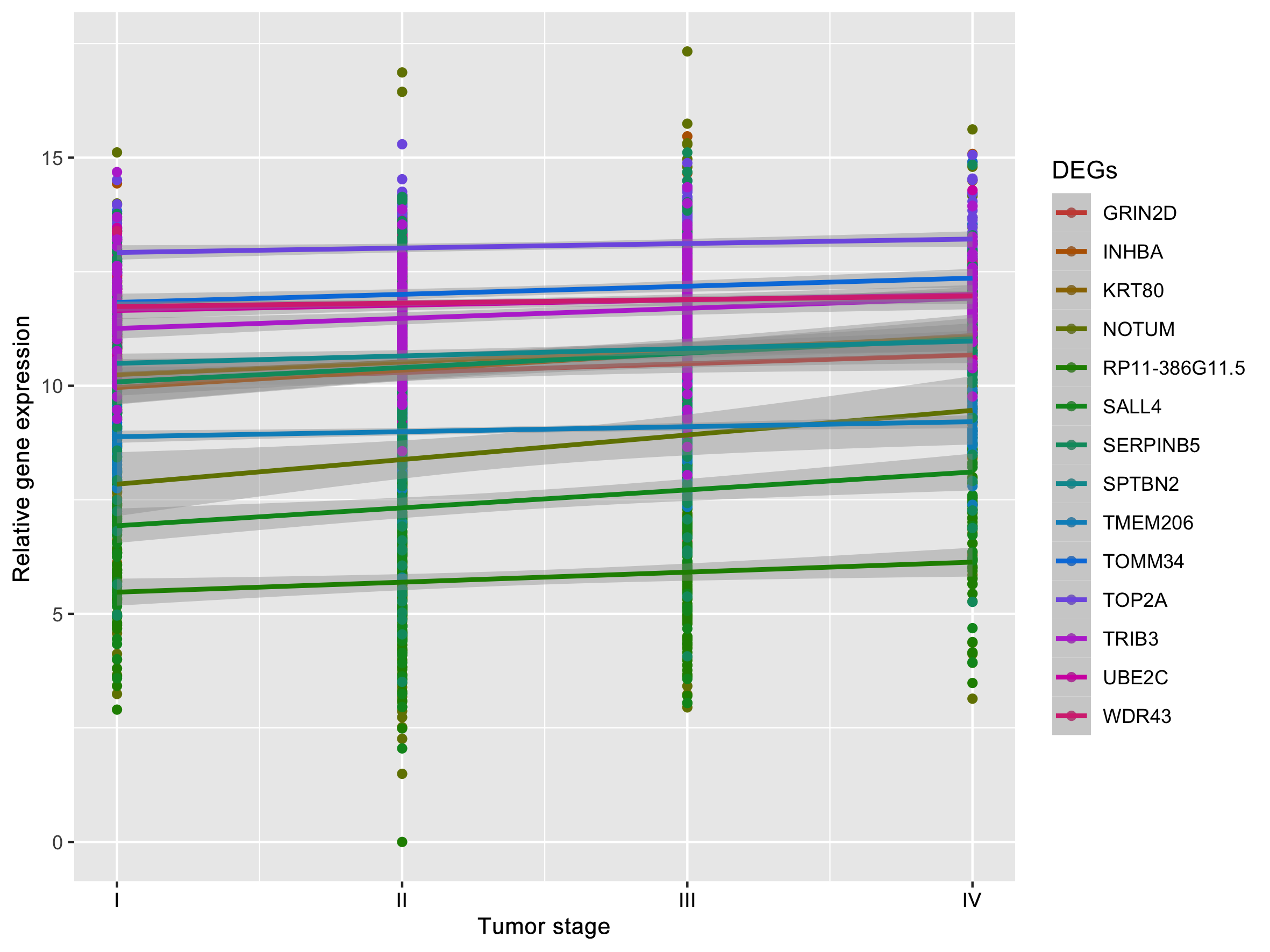

Supplement: Supplementary file 2 — Supplementary Information 2. [file 41598_2022_12780_MOESM2_ESM.tif]

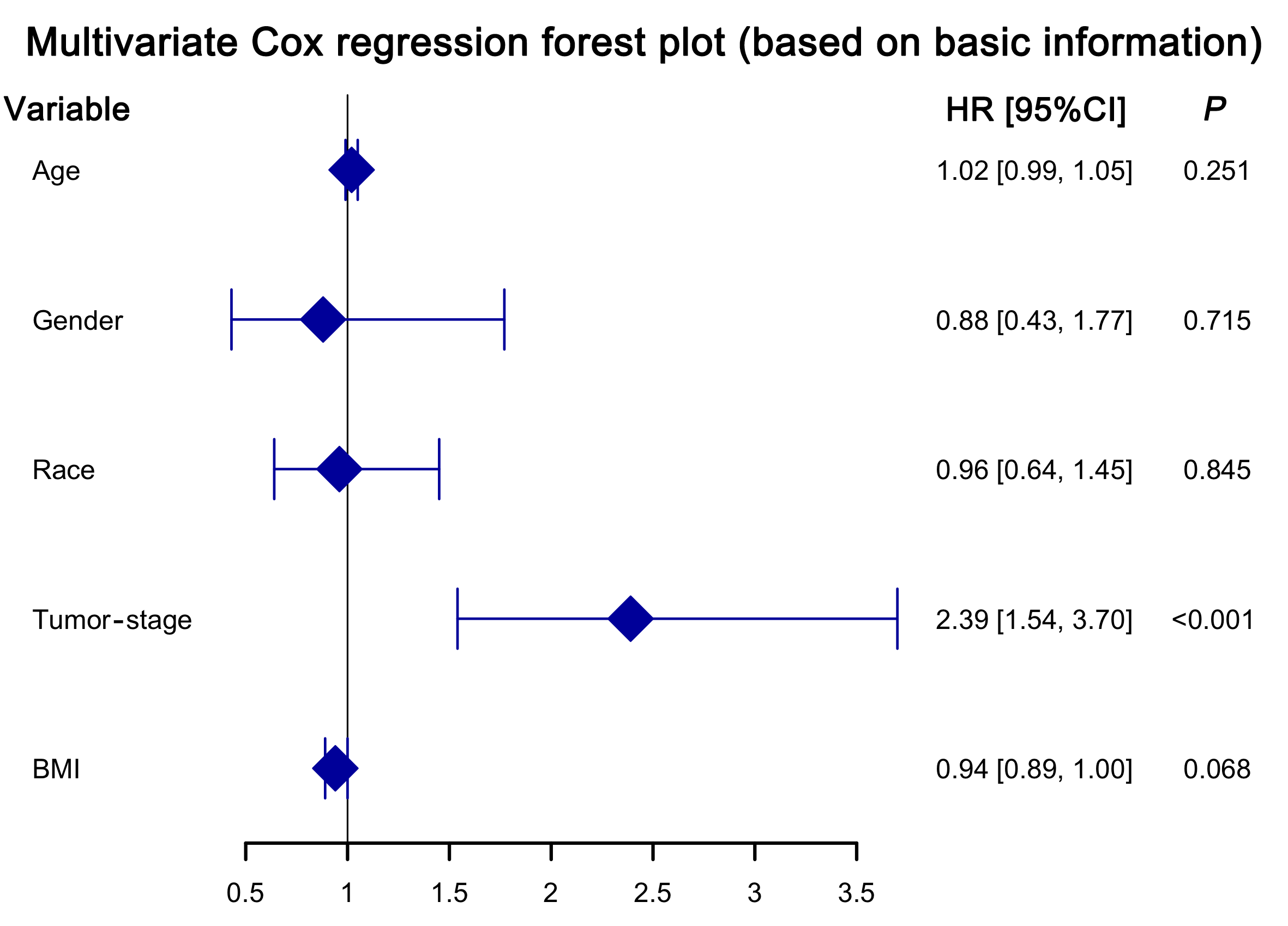

Supplement: Supplementary file 3 — Supplementary Information 3. [file 41598_2022_12780_MOESM3_ESM.tif]
