# Supplementary material for: Identification of gene signatures for COAD using feature selection and Bayesian network approaches
Source: Sci Rep. 2022 May 24;12:8761. doi: 10.1038/s41598-022-12780-7 (PMC9130243; doi:10.1038/s41598-022-12780-7)
Supplement: Supplementary file 4 — Supplementary Information 4. [file 41598_2022_12780_MOESM4_ESM.docx]

**Figure S1.** **PLS-DA plot based on the expression pattern of the 38-genes signatures according of the basic clinical variables.** The analysis was carried out using the “FactoMineR” package for PCA and the “mixOmics” package for PLS-DA in R. Each dot, triangle, and diamond represent a sample.

**Figure S2. Correlation scatter plot of 14 stage-related candidate genes.** The analysis was performed using Pearson correlation in R.

**Figure S3. Multivariate Cox regression forest plot based on basic clinical data.** The analysis was carried out using “survival” and “survminer” packages in R. HR: hazard ratio; CI: confidence interval.
