# Supplementary material for: Identification of gene signatures for COAD using feature selection and Bayesian network approaches
Source: Sci Rep. 2022 May 24;12:8761. doi: 10.1038/s41598-022-12780-7 (PMC9130243; doi:10.1038/s41598-022-12780-7)
Supplement: Supplementary file 5 — Supplementary Information 5. [file 41598_2022_12780_MOESM5_ESM.docx]

**Table S1. Expression information and correlation analysis about the 14 stage-positive related DEGs**

| **DEGs** | **Average expression in normal and tumor samples** | | | | | **Pearson correlation of genes with tumor stage** | |
| --- | --- | --- | --- | --- | --- | --- | --- |
|  | **Normal** | **Tumor** | | | |  |  |
|  |  | **Stage I** | **Stage II** | **Stage III** | **Stage IV** | **Coefficient** | ***P*** |
| GRIN2D | 3.931 | 10.284 | 10.186 | 10.462 | 10.803 | 0.124 | 0.039 |
| INHBA | 5.313 | 9.884 | 10.340 | 10.846 | 10.929 | 0.209 | 0.000 |
| KRT80 | 3.124 | 10.539 | 10.285 | 10.941 | 11.074 | 0.192 | 0.001 |
| NOTUM | 1.274 | 8.523 | 7.993 | 8.871 | 9.888 | 0.154 | 0.010 |
| RP11-386G11.5 | 2.202 | 5.847 | 5.544 | 5.722 | 6.538 | 0.148 | 0.013 |
| SALL4 | 3.625 | 7.145 | 7.114 | 7.928 | 8.008 | 0.206 | 0.001 |
| SERPINB5 | 3.829 | 10.200 | 10.318 | 10.747 | 11.061 | 0.127 | 0.035 |
| SPTBN2 | 6.699 | 10.685 | 10.501 | 10.914 | 10.983 | 0.153 | 0.011 |
| TMEM206 | 6.381 | 8.847 | 9.022 | 9.081 | 9.215 | 0.163 | 0.007 |
| TOMM34 | 8.865 | 11.890 | 11.963 | 12.192 | 12.383 | 0.185 | 0.002 |
| TOP2A | 7.494 | 12.963 | 12.981 | 13.151 | 13.204 | 0.124 | 0.039 |
| TRIB3 | 6.789 | 11.403 | 11.385 | 11.708 | 11.991 | 0.194 | 0.001 |
| UBE2C | 5.568 | 11.682 | 11.759 | 11.848 | 12.052 | 0.120 | 0.046 |
| WDR43 | 9.263 | 11.778 | 11.784 | 11.894 | 11.984 | 0.139 | 0.021 |
