# Supplementary material for: Identification of gene signatures for COAD using feature selection and Bayesian network approaches
Source: Sci Rep. 2022 May 24;12:8761. doi: 10.1038/s41598-022-12780-7 (PMC9130243; doi:10.1038/s41598-022-12780-7)
Supplement: Supplementary file 6 — Supplementary Information 6. [file 41598_2022_12780_MOESM6_ESM.docx]

**Table S2. Univariate and multivariate Cox regression analysis of the 38 genes**

| **Gene features** | **Univariate analysis** | | **Multivariate analysis** | |
| --- | --- | --- | --- | --- |
|  | HR [95% CI] | *P* | HR [95% CI] | *P* |
| MMP7 | 1.07 [0.97, 1.18] | 0.150 |  |  |
| KRT80 | 1.13 [0.94, 1.36] | 0.202 |  |  |
| NOTUM | 1.00 [0.94, 1.08] | 0.932 |  |  |
| TNS4 | 1.03 [0.88, 1.20] | 0.721 |  |  |
| S100P | 0.89 [0.76, 1.04] | 0.128 |  |  |
| SERPINB5 | 0.99 [0.89, 1.09] | 0.821 |  |  |
| GRIN2D | 1.01 [0.86, 1.19] | 0.907 |  |  |
| UBE2C | 1.05 [0.81, 1.35] | 0.715 |  |  |
| RRM2 | 0.94 [0.66, 1.34] | 0.735 |  |  |
| SAPCD2 | 1.16 [0.86, 1.56] | 0.326 |  |  |
| VWA2 | 1.05 [0.88, 1.26] | 0.590 |  |  |
| TPX2 | 0.93 [0.69, 1.25] | 0.624 |  |  |
| WNT2 | 1.04 [0.88, 1.23] | 0.637 |  |  |
| TOP2A | 0.89 [0.65, 1.21] | 0.454 |  |  |
| STRA6 | 1.07 [0.95, 1.21] | 0.253 |  |  |
| OTX1 | 1.03 [0.89, 1.18] | 0.693 |  |  |
| TRIM29 | 0.97 [0.84, 1.12] | 0.655 |  |  |
| INHBA | 1.12 [0.97, 1.29] | 0.134 |  |  |
| SGOL1 | 0.95 [0.68, 1.33] | 0.751 |  |  |
| **TRIB3** | 1.25 [0.99, 1.56] | **0.058** | 1.21 [1.04, 1.48] | 0.110 |
| TRIP13 | 1.05 [0.73, 1.51] | 0.800 |  |  |
| TESC | 0.94 [0.84, 1.05] | 0.276 |  |  |
| ZWINT | 0.99 [0.70, 1.41] | 0.973 |  |  |
| SALL4 | 1.08 [0.94, 1.24] | 0.277 |  |  |
| SPTBN2 | 1.04 [0.81, 1.34] | 0.768 |  |  |
| RP11-386G11.5 | 1.05 [0.88, 1.25] | 0.622 |  |  |
| TMEM97 | 1.05 [0.77, 1.43] | 0.742 |  |  |
| TOMM34 | 1.09 [0.84, 1.42] | 0.524 |  |  |
| TMEM206 | 0.98 [0.66, 1.45] | 0.914 |  |  |
| WDR43 | 1.01 [0.64, 1.60] | 0.953 |  |  |
| TMEFF2 | 1.10 [0.92, 1.31] | 0.318 |  |  |
| **STMN4** | 1.15 [0.98, 1.35] | **0.080** | 1.24 [1.04, 1.48] | **0.017** |
| **FAM135B** | 0.85 [0.71, 1.02] | **0.087** | 0.81 [0.67, 0.98] | **0.032** |
| GLP2R | 0.93 [0.81, 1.06] | 0.281 |  |  |
| RERGL | 1.08 [0.95, 1.22] | 0.257 |  |  |
| SFRP5 | 1.08 [0.97, 1.21] | 0.140 |  |  |
| SCN7A | 1.04 [0.95, 1.14] | 0.343 |  |  |
| PLP1 | 1.00 [0.91, 1.11] | 0.968 |  |  |

The univariate and multivariate Cox regression analysis were performed using “survival” and “survminer” packages in R. HR: hazard ratio; CI: confidence interval.
